# N. Y. Annalist

**Published:** 1847-06

**Authors:** 


*N. Y. Annalist.* — Our much respected contemporary, the Annalist, in a deservedly complimentary notice of the May No. of the N. Y. Journal of Medicine remarks, "this (the N. Y. Journal) is the only journal of any size in this city or state." We beg to remind the Editor of the Annalist that the Buffalo Medical Journal in point of size falls but a few pages short of the New-York Journal, although we are very far from assuming that in other respects it will bear comparison with that valuable periodical. From the well-known courtesy of the accomplished Editor of the Annalist, we do not doubt that he will cheerfully correct the error into which he had inadvertently fallen.

